# Implementation of isoniazid preventive therapy in people living with HIV in Zambia: challenges and lessons

**DOI:** 10.1186/s12889-019-7652-x

**Published:** 2019-10-22

**Authors:** Mary Kagujje, Muhau L. Mubiana, Eugenia Mwamba, Monde Muyoyeta

**Affiliations:** Centre for Infectious Disease Research in Zambia, Plot # 34620, Off Alick Nkhata Road between ERB and FAZ, Mass Media, P. O Box 34681, 10101 Lusaka, Zambia

**Keywords:** Tuberculosis, Prevention, HIV, Chemoprophylaxis, Health policy

## Abstract

**Background:**

Uptake of Isoniazid Preventive Therapy (IPT) among People Living with HIV in Zambia has continued to be low despite various evidence for its added benefit in reducing TB incidence and mortality when taken with antiretroviral therapy. In 2017, only 18% of People Living with HIV newly enrolled in care were initiated on IPT in Zambia.

**Main text:**

Various challenges including policy and management level factors, supply chain factors, health worker perceptions about IPT, monitoring and evaluation factors and limited demand creation activities have constrained the scale up of IPT in Zambia. Lessons that have been learnt while addressing the above challenges are shared and they can be applied by government ministries, project managers, public health specialists to strengthen IPT activities in their settings.

**Conclusion:**

Zambia has both a high burden of TB and HIV and without preventing new cases of TB from reactivation of latent TB infection, it will be difficult to control TB. All stakeholders involved in prevention of TB among PLHIV need to commit to addressing the challenges limiting scale up of IPT.

## Background

It is estimated that almost one quarter of the world’s population has latent TB and 5–10% of these will develop active TB during their lifetime [1, 2]. Weak immunological status is the most important risk factor for progression from latent to active TB; People Living with Human Immunodeficiency Virus (PLHIV) are 21 times more likely to develop active TB than those without HIV [2]. Despite increased access to anti-retroviral therapy (ART), with wide scale implementation of test and treat, TB has continued to be the leading cause of mortality among PLHIV [3].

ART alone reduces the incidence of TB by up to 65% [4] while a combination of Isoniazid Preventive Therapy (IPT) and ART reduces the overall incidence and mortality from TB by up to 90% [5, 6]. Zambia is a high burden TB country with a case detection rate of only 58% [2]; this is suggestive of ongoing transmission and high rates of latent TB infection. It also has a high HIV prevalence of 12.3% among adults [7] and this represents a big pool of the population at risk for active TB. Prevention of active TB among PLHIV, is not only critical to meeting the World Health Organisation (WHO) End TB targets but also important for improved outcomes of patients on HIV treatment.

IPT was piloted in Zambia in 2013 through the Centre for Disease Control and Prevention (CDC) supported project on intensified case finding, IPT and infection control (3Is) which was in 18 health facilities in Lusaka, Central, Southern and Copperbelt provinces. In July 2014, Zambia published its first guidelines on the 3Is titled “Managing Tuberculosis in the HIV setting in Zambia” which recommended daily isoniazid for 6 months for the treatment of latent TB among PLHIV and children below five years that are contacts to bacteriologically confirmed TB cases [8]. In 2016, IPT was then rolled out to the other provinces and health facilities across Zambia. In the 2016 WHO report, which was the first report including data on TB preventive services, 66% of the PLHIV newly enrolled in care in Zambia were initiated on IPT [9]. This data was reported from the facilities that were supported by the 3Is project. In the 2017 and 2018 WHO reports, only 15 and 18% of the PLHIV newly enrolled in care were initiated on IPT respectively [2, 3]. A CDC funded project being implemented by Centre for Infectious Disease Research (CIDRZ) across 4 of the 10 provinces in Zambia; Lusaka, Eastern, Western and Southern provinces; reported that only 30% of the PLHIV newly enrolled into care were initiated on IPT during the periods October 2016 to September 2017 and October 2017 to March 2018 (Unpublished observation, CIDRZ TB Department annual report 2017 and semi-annual report 2018). Between October 2016 and September 2017, the project supported 13 hospitals and 137 clinics whilst between October 2017 and March 2018, it supported 18 hospitals, 453 clinics and 138 health posts.

Uptake of IPT among PLHIV newly enrolled in care has been slow in most high TB burden countries. In fact, of the 12 high burden countries that reported data to WHO, coverage ranged between 2.4 and 73.0%. This paper shares specific challenges that can be encountered by government ministries, project managers, public health specialists and funding agencies in roll out and scale up of IPT. It also provides important lessons that can either be used to avoid or to address the challenges.

## Main text

### Challenges

#### Policy and management level factors

The Global Fund requires TB and HIV joint programming and emphasizes synergistic implementation of TB and HIV activities [10]. In Zambia planning, implementation, monitoring, evaluation and reporting on IPT is primarily driven by the National TB and Leprosy Program (NTLP) and to a lesser extent supported by the HIV program.

Previously, both programs provided guidelines for use of IPT among PLHIV and there were minor differences in the recommendations from the 2 programs for example: 1) The TB program recommended use of IPT among all PLHIV while the HIV program, due to prevailing logistical challenges surrounding available stock of IPT, recommended use of IPT among PLHIV newly enrolled in care and 2) the HIV guidelines recommended that IPT must be given with Vitamin B6 while the guidelines from the TB program were silent on the use of Vitamin B6 [8, 11]. The discrepancy in the previous guidelines was addressed in the new guidelines on TB preventive therapy that were launched in March 2019. The lack of TB/HIV coordinating body complicated decision making and efforts for scale up.

At project management level, the focus in rolling out IPT was on clinics and hospitals and there was a general delay in rolling out IPT program to the health posts. Because the health posts collectively serve a big volume of PLHIV, this delay significantly contributed to the low coverage of IPT in Zambia.

#### Supply chain factors

In the initial stages of IPT implementation, ordering of IPT by the facility was based on the number of TB cases reported. However, the commodity was used for patients without active TB and projections had also been made based on the same. As a result, the country recorded expiries of the commodity due to a pseudo low demand from the facilities. A decision to include isoniazid on the ARVs report and requisition and have requisitions placed by the ART clinic was made and this enabled most of the facilities to pull the desired stocks of the drug from the central warehouse.

Challenges with INH stock levels have continued in some facilities due to: 1) compromised quality of reports and requisitions which cause some facilities to stock out while others are over stocked such that if redistributed, most facilities that were previously understocked would be adequately stocked, 2) rapid scale up of test and treat with a resultant drastic increase in the number of people eligible for IPT resulting in stock out of INH and 3) rapid transition of mobile ART services to static ART services in health posts which resulted into rationing of the quantities given to clinics in order to accommodate their satellite health posts.

Because the Zambia consolidated guidelines for prevention and treatment of HIV recommend concomitant use of INH with Vitamin B6 to prevent side effects among PLHIV, stock outs of vitamin B6 adversely affect INH uptake due to low prescribing and low dispensing. Vitamin B6 stock out has been a repetitive occurrence and this is both due to inadequate forecasting and accountability; INH is on the ARV logistics system while pyridoxine is on the essential medicines logistics system.

#### Health worker perceptions about IPT

In a file review conducted by the Pharmacy department of CIDRZ only 56 (50%) of the 113 eligible patients had IPT prescribed and only 37(66%) of the 56 that had a prescription received IPT (Unpublished observation). Fears and misconceptions among health workers in other parts of the world have also been observed in Zambia [12]. The commonest fears are: 1) use of IPT promotes isoniazid mono-resistance since symptom screening alone is not sufficient to rule out active TB, 2) the protective effect of IPT is minimized due to ongoing exposure to TB since Zambia is a high burden country 3) increased risk of side effects among PLHIV. These conceptions usually hinder both prescription and dispensation of IPT especially in the secondary and tertiary level health facilities.

Since most IPT activities are driven through donor funding with partner support, ownership of the IPT program at health facility level has continued to be a challenge. Often times, IPT scale up is perceived as a partner priority and not a Ministry of Health priority.

#### Monitoring and evaluation factors

Among the electronic health record systems implemented by Zambia are SmartCare which is used as patients’ record and the electronic Logistics Management Information System (eLMIS) which is used as logistics or inventory records. These two systems are not interoperable, but even if they were, they wouldn’t capture all the information in the IPT register. A paper based IPT register is used for monitoring IPT implementation. Facility staff especially Pharmacy are required to use SmartCare for dispensations, eLMIS to update inventories and paper-based information systems leading to compromised data capturing.

Incomplete documentation in the IPT register, which is usually placed at the pharmacy, is a persistent challenge in some health facilities. It is characterized by some or all of the following: 1) failure to enter a patient in the IPT register when they start treatment, 2) failure to update the register when a patient receives refills and 3) failure to document the final outcome of the patient. This is especially common in health facilities that attend to a high volume of clients because the pharmacy personnel need to create a balance between completely documenting in the various registers and ensuring that they do not increase patients’ waiting time. The poor documentation as a result makes it difficult to: 1) understand the actual coverage of IPT, 2) do quantification for adequate stock levels and 3) monitor the effectiveness of IPT.

Data collection processes also further compromise the quality of IPT data. For the purposes of efficiency, health facilities may have more than one IPT register to cover different departments where IPT is offered, e.g. Maternal Child Health clinics, TB clinic and ART clinic. Health posts also have their own registers. It has been observed that some registers are missed out during data collection. This problem is compounded on dependency on the paper-based data recording tools.

In addition, until recently, data review meetings at all levels didn’t cover IPT indicators which perpetuated the lack of accountability in scaling up uptake of IPT.

#### Limited demand creation activities

Demand creation activities for IPT are mainly done as part of demand creation for the 3Is. However, generally speaking, this demand creation has focused more on intensified case finding in comparison to the other components of the 3Is. To date, there is a lot of Information, Education and Communication (IEC) materials on intensified case finding but no locally adapted or developed IEC material on IPT.

This creates a challenge for the health workers that are willing to prescribe IPT to eligible clients because they have to spend a lot of time explaining to the clients why they need IPT and its possible side effects; this is more difficult for health staff working in facilities that have a high patient health worker ratio.

### Lessons learnt

As per the recommendation from the Global Fund, there should be an active TB/HIV coordinating body to oversee IPT implementation in Zambia. This will facilitate and enhance: 1) harmonization of guidelines on IPT, 2) integrated planning for scale up of TB/HIV care to minimize duplication of effort, 3) management of the IPT supply chain in relation to the expected PLHIV newly enrolled in care, 4) buy in and accountability from the health workers in HIV clinics, and ultimately 5) quality of HIV care.

For a health facility supporting satellite ART sites, roll out of IPT should only be considered complete when IPT activities are being implemented in all its supported health posts. In addition, staff from the health facility must be involved in the process. This not only allows for continuous mentorship but also facilitates better quantification of IPT needs.

There was limited engagement of pharmacy personnel at all levels in the roll out IPT no wonder the scale up process has suffered multiple supply chain challenges. Deliberate investment in training and mentorship on IPT guidelines and supply chain management of IPT and related commodities, especially in facilities where there is task shifting to non-pharmaceutical staff, needs to be made.

Forecasting and quantification of isoniazid and Vitamin B6 should involve all the relevant stakeholders to allow for adequate stock levels that are sufficient for the eligible populations. To improve accountability of stock management, the supply chains for the two drugs should be synchronized either by putting isoniazid and vitamin B6 on the same logistics system or by co-packaging the two drugs.

Clinicians need to be engaged on IPT during their continuous medical education sessions in order to demystify IPT; sufficient information on prevention, early detection and management of the side effects of IPT should be provided. Where possible IPT should be added to the pre-service training. Peer to peer mentorship and exchange visits between clinicians and pharmacists in well performing and poorly performing sites is also a useful strategy.

Investment in obtaining complete documentation either through use of more robust interoperable electronic information systems or through task shifting of documentation in paper-based registers to community health workers as needed should be made in order to obtain quality data which can be used to guide decisions and program improvement.

A data collection log with the capacity to flag health facilities, including health posts, and specific departments in a health facility with missing data on IPT must be implemented to facilitate completeness of data.

There should be continuous sharing of performance data between program managers and the health facilities to motivate quality improvement in IPT implementation.

HIV data review meetings should routinely include IPT indicators for PLHIV.

Demand creation activities for IPT should take a three-pronged approach, that is 1) targeted messages specific to IPT 2) incorporation into ART adherence counselling and health talks and 3) continued integration into demand creation for intensified TB case finding and infection control. In consultation with HIV control programs and health promotions programs, IEC materials on IPT should also be developed.

## Conclusion

Zambia has both a high burden of TB and HIV and without preventing new cases of TB from reactivation of latent TB infection, it will be difficult to control TB. The Ministry of Health and other stakeholders involved in prevention of TB among PLHIV need to commit to addressing the challenges limiting scale up of IPT. Investment in prevention of active TB will not only contribute to ending TB but will also improve the quality of HIV care, improve outcomes of HIV treatment and promote cost effectiveness and equity in health.

The challenges observed in Zambia are not unique to it as they have been observed in other settings [12, 13]. The solutions can hence be applied or adapted to various settings.

## Data Availability

Not applicable.

## Abbreviations

ART: Anti-retroviral therapy
CDC: Centre for Disease Control and Prevention
CIDRZ: Centre for Infectious Disease Research in Zambia
eLMIS: electronic Logistics Management Information System
HIV: Human Immunodeficiency Virus
IEC: Information, Education and Communication
IPT: Isoniazid Preventive Therapy
PEPFAR: United States President’s Emergency Plan for Aids Relief
PLHIV: People Living with Human Immunodeficiency Virus
TB: Tuberculosis
UNAIDS: United Nations Program on HIV/AIDS
WHO: World Health Organisation
